# An evidence review of the association of immune and inflammatory markers with obesity-related eating behaviors

**DOI:** 10.3389/fimmu.2022.902114

**Published:** 2022-07-15

**Authors:** Ying Meng, Amber Kautz

**Affiliations:** ^1^ School of Nursing, University of Rochester, Rochester, NY, United States; ^2^ Department of Public Health Sciences, University of Rochester Medical Center, Rochester, NY, United States

**Keywords:** eating behavior, immune markers, cytokine, obesity, inflammatory marker

## Abstract

**Background:**

Eating behaviors contribute to disproportionate energy intake and are linked to the development of obesity. Animal studies support the role of inflammatory cytokines and chemokines in the regulation of obesity-related eating behaviors and offer a potential target to combat obesity through the modulation of inflammation. However, more complex eating behaviors are present in humans, and their relationships with immune/inflammation markers are unclear. The present study reviewed current literature to synthesize the evidence on the association of immune/inflammation markers with obesity-related eating behaviors in humans.

**Methods:**

A systematic search of three electronic databases yielded 811 articles, of which 11 met the inclusion criteria.

**Results:**

The majority of the included studies (91%) were either case-control or cross-sectional studies. A variety of immune/inflammation markers and obesity-related eating behaviors have been assessed in the chosen studies. Three out of four studies identified a positive relationship between C-reactive protein (CRP)/high-sensitivity CRP and loss of control eating. Other inflammatory markers that potentially have a positive relationship with obesity-related eating behaviors include fractalkine and fibrinogen. Additionally, immune molecules, including interferon gamma (INF-γ), interleukin (IL)-7, IL-10, and α-melanocyte-stimulating hormone-reactive immunoglobulin G (α-MSH/IgG) immune complex, may have negative associations with obesity-related eating behaviors. However, most findings were identified by single studies.

**Conclusion:**

Limited studies have been conducted in humans. Current evidence indicates a potential bi-directional relationship between inflammatory/immune markers and obesity-related eating behaviors. Additional studies with sophisticated research design and comprehensive theoretical models are warranted to further delineate the relationship between immune/inflammation markers and obesity-related eating behaviors.

## Introduction

The prevalence of obesity has continued to rise. Over 42% of adults and 19% of children are currently affected by obesity in the United States (1, 2). Eating behaviors, such as eating in the absence of hunger, disinhibited eating, and excessive intake of dietary fat, contribute to disproportionate energy intake and have been linked to the development of obesity (3). Strategies targeting obesity-related eating behaviors may be a key approach to alleviate the obesity epidemic, and comprehensive understanding of the underlying mechanisms is crucial to develop effective interventions.

A growing body of evidence has indicated that immune and inflammatory molecules play a role in the regulation of eating behaviors (4–7). It has long been observed that various disease states can lead to reduced appetite and food intake (8). Several cytokines, such as tumor necrosis factor (TNF)-α, interleukin (IL)-1α, IL-1β, IL-6, IL-7, IL-18, have been found to suppress food intake in animals and potentially in humans (4, 7, 9–12). In addition to suppressing appetite, alterations in immunological function may also be involved in the pathogenesis or perpetuation of obesity-related eating behaviors. In rodent models, deficiency in IL-6, IL-1 type I receptor, and IL-18 resulted in hyperphagia and obesity (6, 13, 14). Intracerebroventricular administration of IL-6 and IL-1β reduced sucrose preference and food intake (7, 15). Alleviation of hypothalamic inflammation and deficiency in interferon gamma (IFN-γ) also reduced food intake and diet-induced obesity (16–18). Hence, it is hypothesized that dysregulation of the immune system may initiate or exacerbate appetite and food reward signals and contribute to the development or perpetuation of obesity-related maladaptive eating behaviors.

Previous studies in animals supported the role of cytokines in the regulation of obesity-related eating behaviors (6, 7, 13–18). However, there has been limited research exploring the involvement of immune molecules in modulating obesity-related eating behaviors in humans which are more complex than rodent models (19). Therefore, this review was conducted to synthesize the evidence on the association between immune markers and obesity-related eating behaviors in humans.

## Methods

To identify relevant publications, systematic searches were conducted using the following electronic databases: PubMed, Web of Science, and CINAHL. We used a variety of search terms including “eating” or “feeding” combined with “obesity” then crossed with “immune” or “inflammation” or “cytokine”. The searches were limited to “human” studies, and articles were restricted to English language. Primary research studies published through May, 2021 were included if they fulfilled the following criteria: 1) the relationship between immune/inflammatory molecules and eating behaviors related to obesity was examined; 2) to expand the number of articles included, articles comparing anorexia nervosa (AN) with obesity were also included, and AN was considered an eating behavior opposite of those related to obesity; 3) articles examining brain regions related to eating behavior regulation were included, and the brain regions were used as proxies for eating behaviors. Studies were excluded if 1) they discussed the links between inflammatory markers with fasting or intake of single foods, nutrients, or dietary patterns; or 2) they examined local infections, such as gastric infection by *Helicobacter pylori*; or 3) they assessed leptin instead of inflammatory markers, as leptin is commonly classified as an adipokine; or 4) they were conducted in patients with cancers.

**Figure 1:**
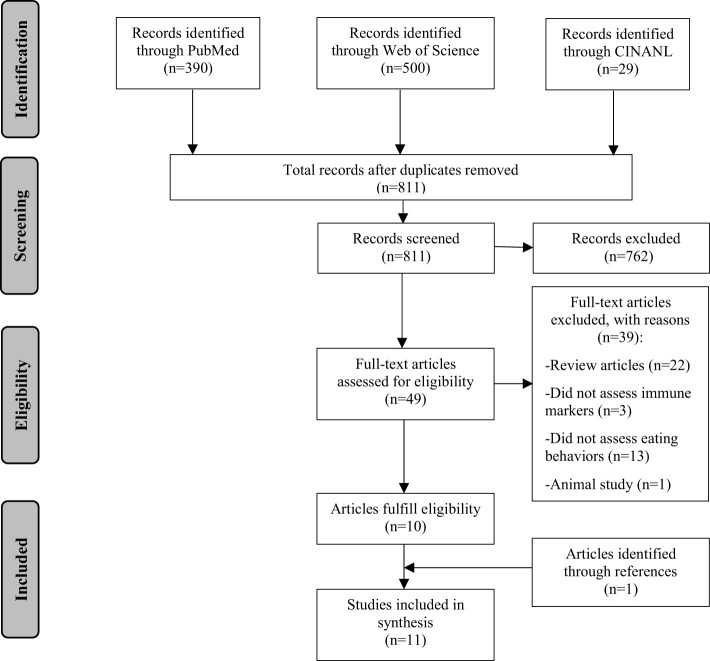
Literature search flow chart.

**Figure 1** presents the flow of the literature search. A total of 811 articles were screened. Two reviewers (YM and AK) independently screened all titles and abstracts to identify potential articles (n=49). Full texts were extracted and reviewed by the two reviewers. Of these articles, ten articles fulfilled the eligibility criteria. References of the identified articles were further screened. One additional article was identified. A total of 11 articles were included in this review.

## Results

### Study characteristics

Of the eleven included articles (**Table 1**), five studies assessed a variety of obesity-related eating behaviors, including loss of control eating, hunger, binge eating, and emotional eating. Five studies compared eating disorders with obesity. One study examined brain regions related to the regulation of food intake. A variety of cytokines, chemokines, immunoglobulins and inflammatory markers have been assessed in these studies. With regards to study designs, six studies were case-control studies, four studies were cross-sectional studies, and one study utilized a quasi-experimental design. Six studies were conducted in Europe, three in the USA, one in Brazil, and one in China. The sample sizes ranged from 32 to 194. Five studies included more than or equal to100 participants. Four studies recruited children and adolescents, and the other studies recruited adults or a mixture of adolescents and adults. Five studies only conducted assessments with female participants.

**Table 1:** Included studies.

Article	Study design	Sample size, Nation,Age, and Gender	Eating behaviors	Immune markers/cytokines	Variables adjusted	Summary of main findings
Capuron, et al. (20)	Quasi-experi-mental (pretest-posttest design)	N=101; France; Age 37.8±11.2; Female 100%	TFEQ51 (cognitive restraint, disinhibition, hunger)	IL-6, hsCRP	Age and diabetes status	At baseline, IL6 and hsCRP was negatively associated with hunger scores. But these associations were not significantly after adjusting for BMI. Decreases in IL6 and hsCRP 1-year post gastric surgery were associated with reduction in cognitive restraint scores after adjusting for age, diabetes and variation in BMI.
Caroleo, et al. (21)	Case-control	N=90; Italy; Age 36.7±13.2; Female 69%	AN, BED, obesity without BED, and normal-weight healthy controls, grazing, emotional eating, craving for carbohydrates, sweet eating, post-dinner eating, night eating, binge eating, hyperphagia, social eating, skipping meals, reducing portions	IL-1α, IL-1β, IL-2, IL-4, IL-6, IL-8, IL-10, IFN-γ, TNF-α, MCP-1	Beck Depression Inventory scores, sex and age	IL-1α was lower in AN patients or patients with obesity with or without BED than in healthy controls, while IL-1α was not influenced by BMI or depression. IL-10 was higher in AN than in healthy controls. IL-10 was lower in obesity groups compared to normal weight group. IFN-γ was higher in AN patients compared to healthy controls, and BED and obesity without BED individuals. Night-eating was positively associated with IL-8. IL-10 was positively related to post-dinner eating and negatively related to sweet eating.
Cazettes, et al. (22)	Case-control	N=63; USA; Age 58.7±7.7 in overweight and obese group; Female 51%	Brain regions related to food intake: lateral and medial orbitofrontal cortex (OFC), hippocampus volume, gray matter	Fibrinogen	Age and hypertension	Fibrinogen was significantly associated with smaller lateral OFC volume in the overweight and obese group. Among lean individuals, higher fibrinogen levels were associated with lower apparent diffusion coefficient (ADC) (less interstitial fluid) in the left prefrontal, right parietal and left occipital lobes. Among individuals with excess weight, elevations in fibrinogen concentration were associated with increased ADC (greater interstitial fluid) in both amygdala and the right parietal lobe.
Germain, et al. (23)	Case-control	N=51; France; Age 21.6±1.5 in AN-R and 27±2.1 in OB; Female 100%	AN restrictive type (AN-R), AN restrictive type after recovery (AN-R rec), anorexia nervosa bulimic type (AN-BP), constitutional thinness (CT), bulimia nervosa (BN), healthy obese (OB), and control subjects	IL-7	Not specified	24-hour mean levels of IL-7 were significantly lower in AN subtypes when compared to controls, BN, and CT. IL-7 was lower in AN-BP than in AN-R and AN-R rec. IL-7 in BN did not differ from controls. IL-7 in CT was also similar to controls. IL-7 was significantly decreased in OB compared to controls.
Lofrano-Prado, et al. (24)	Case-control	N=32; Brazil; Age 14-19; Female: Not specified	Binge eating scale (BES) Bulimic Investigation test Edinburgh (BITE)	TNF-α	α-MSH and insulin	TNF-α had a positive correlation with BES and BITE. α-MSH, insulin and TNF-α provided the best model to explain the variability in BES and BITE.
Lucas, et al. (25)	Case-control	N=158; France and Estonia; Age 47.2±16.3 for hyperphagia obesity; 18.6±4.9 restrictive AN; 30.6±11.6 BED; Female 100%	Hyperphagic obesity (OB), AN, BN, BED, and healthy controls	α-MSH/IgG immune complex	Not specified	Both association and dissociation rates of α-MSH-reactive IgG were decreased in OB patients. Dissociation rate was increased in BN and BED groups. Plasma levels of α-MSH-reactive IgG were low in OB patients but high in BN compared to controls. The MC4R receptor binding affinity, internalization, and MC4R activation of α-MSH/IgG immune complex was decreased in OB patients. BN had higher membrane location compared to AN. Higher internalization rate of the immune complex was found in AN group. The anorexigenic effect of α-MSH/IgG immune complex was reduced in OB at 120 minutes during refeeding. Several alternative epitopes were present in OB IgG, overlapping with the C-terminal and with the pharmacophone. In contrast, reduced binding of the central α-MSH part was present in IgG from ED patients.
Raymond, et al. (26)	Case-control	N=93; USA; Age 16-50; Female 100%	AN, OB, BN, normal weight control	IL-1α, IL-6, TNF-α, IFN-γ, TGF-β	None due to no correlations of cytokine levels with BMI, age, time of venipuncture day, depression or anxiety.	ConA-stimulated IFN-γ by cultured PBMC for AN participants were significantly higher than those of normal weight control group. Stimulated PBMC from the obese participants produced significantly higher levels of IL-6 than the BN or normal-weight control groups. IL-1α production by stimulated cells from the obese group was significantly elevated in comparison to the control group. No group differences were detected in the stimulated production of TGF-β or TNF-α. There were no differences in the levels of any of the five cytokines produced by unstimulated cultured PBMC across the four groups.
Sayin, et al. (27)	Cross-sectional	N=100; Turkey; Age 12-17; Female 64%	TFEQ21 (emotional eating and uncontrolled eating)	CRP	Sex, age, BMI	CRP positively correlated with EE and UCE. EE was significantly associated with CRP adjusting for age, gender, or BMI, or UCE.
Shank, et al. (28)	Cross-sectional study	N=194; USA; Age 8-18; Female 64%	Loss of control eating (LOC) in the past month	hsCRP	Treatment, sex, race, fat mass, height, Tanner stage, depressive symptoms, eating psycho-pathology	Presence of LOC eating was significantly associated with higher hsCRP concentration. An increase in the number of LOC eating episodes in the past month was significantly associated with higher hsCRP concentration.
Succurro, et al. (29)	Cross-sectional	N=115; Italy; Age 36.8±12.7 BED obese group 41.8±12.8 non-BED obese group; Female 65%	BED	hsCRP, WBC	Age, sex and BMI	Binge eating disorder obese had higher levels of hsCRP and WBC counts (p<0.01) after adjusting for BMI or age and sex compared to non-BED obese group.
Zhang, et al. (30)	Cross-sectional	N=96; China; Age 11-18; Female 100%	AN, healthy control and OB group	FKN	BMI	Serum FKN levels in the AN group were significantly lower than in the healthy control and OB groups. Serum FKN levels were significantly higher in OB compared to healthy control. Serum FKN per BMI levels was significantly higher in AN group compared to levels in the healthy control and OB groups.

TFEQ is three factor eating questionnaire. AN represents anorexia nervosa; BN represents bulimia nervosa; BED represents binge eating disorder; OB represents individuals with obesity; CT represents constitutional thinness. IL is interleukin; hsCRP is high sensitive C-reactive protein; IFN-γ is interferon-gamma; TNF-α is tumor necrosis factor-alpha; MCP-1 is monocyte chemoattractant protein-1; TGF- β is transforming growth factor beta; α-MSH/IgG is α-melanocyte-stimulating hormone reactive IgG; MC4R is melanocortin-4-receptor; WBC is white blood cell; and FKN is fractalkine.

### Descriptive synthesis of main findings

Four studies examined the relationship between the C-reactive protein (CRP) or high sensitivity-CRP (hsCRP) and eating behaviors, including uncontrolled eating, binge eating, hunger, emotional eating and cognitive restraint eating (**Table 2**). Two of these studies with relatively large sample sizes reported positive associations of hsCRP with loss of control eating or binge eating after controlling for covariates (28, 29). Particularly, these two studies adjusted for adiposity including fat mass or body mass index (BMI) in their models. Another study also reported a positive correlation between CRP and uncontrolled eating (27). Capuron et al. (20), however, did not find a significant association between hsCRP and disinhibition (20). The eating behavior measurements varied among studies, which may account for the inconsistency of findings. Other than disinhibited eating, Sayin et al. (27) also reported a positive association between CRP and emotional eating after controlling for BMI. Capuron et al. (20) found that reduction in hsCRP one-year post bariatric surgery was associated with decrease in cognitive restraint eating.

**Table 2:** Results based on individual immune/inflammation markers.

Article	Eating behaviors	hsCRP	CRP	IL-1α	IL-6	IL-7	IL-8	IL-10	INF-γ	TNF-α	Fibrinogen	α-MSH/IgG immune complex (IC)	Fractalkine	WBC
Capuron et al. (20)	Cognitive restraint	“+” after adjusting for age, diabetes and BMI.			“+” after adjusting for age, diabetes and variation in BMI.									
	Disinhibition	“NS”			“NS”									
	Hunger	At baseline, “-”; “NS” after adjusting for BMI			At baseline, “-”; “NS” after adjusting for BMI									
Shank, et al. (28)	Loss of control (LOC) eating	“+” with presence of LOC eating and the number of LOC episodes.												
Succurro et al (29)	BED-OB vs non-BED-OB	“+” after adjusting for BMI												Higher WBC after adjusting for BMI
Sayin, et al. (27)	Emotional eating		“+” adjusting for age, gender, or BMI, or UCE.											
	Uncontrolled eating (UCE)		“+”											
Caroleo et al. (21)	AN vs NWC			Lower than HCs, not influenced by BMI or depression.	“NS”		“NS”	Higher than NWC	Higher than the other groups.	“NS”				
	BED vs NWC			Lower than HCs, not influenced by BMI or depression.	“NS”		“NS”	“NS”		“NS”				
	OB without BED vs NWC			Lower than HCs, not influenced by BMI or depression.	“NS”		“NS”	Lower than NWC.		“NS”				
	Other eating behaviors, such as emotional eating, craving, grazing, etc.			“NS”	“NS”		“+” with night-eating	“+” with post-dinner eating; “-” with sweet eating.	“+” with long fasting	“NS”				
Raymond, et al. (26)	AN vs NWC			“NS”	“NS”				Produc-tion by stimulated PBMC were higher than NWC	“NS”				
	OB vs NWC			Production by stimulated PBMC elevated than NWC	Stimulated PBMC produced higher levels than NWC.				“NS”	“NS”				
Germain et al. (23)	AN subtypes vs NWC					24-hour mean levels lower than controls								
	BN vs NWC					“NS”								
	CT vs NWC					“NS”								
	OB vs NWC					Decreased than controls								
Lofrano-Prado et al. (24)	BES									“+”				
	BITE									“+”				
Cazettes et al. (22)	Brain regions related to food intake										Associated with smaller lateral OFC volume in the overweight and OB group. Among individuals with excess weight, + with increased ADC in both amygdala and the right parietal lobe.			
Lucas et al. (25)	AN vs HC											Higher internalization rate of IC		
	BN vs HC											Plasma level of IC was high and dissociation rate was increased.		
	BED vs HC											Increased dissociation rates of IC		
	Hyperphagic OB vs HC											Decrease in the association and dissociation rates of IC; α-MSH-reactive IgG were lower; The receptor binding affinity, internalization, and activation by IC was decreased;		
Zhang et al. (30)	AN vs HC												FKN per BMI levels was higher in than HC and OB groups.	
	OB vs HC												levels were higher than HC	

“+” indicates Positive association; “-” indicates negative association; “NS” indicates non-significant. LOC is loss of control eating. UCE is uncontrolled eating. AN represents anorexia nervosa; BN represents bulimia nervosa; BED represents binge eating disorder; OB represents individuals with obesity; CT represents constitutional thinness; NWC and HC represents normal/healthy weight controls. BES is Binge eating scale; BITE is Bulimic Investigation test Edinburgh. IL is interleukin; hsCRP is high sensitive C-reactive protein; IFN-γ is interferon-gamma; TNF-α is tumor necrosis factor-alpha; α-MSH/IgG is α-melanocyte-stimulating hormone reactive IgG; WBC is white blood cell. IL-1β, IL-2, IL-4, transforming growth factor beta (TGF-β), and monocyte chemoattractant protein-1 (MCP-1) was assessed, but no significant results were identified.

Two studies compared IL-1α levels among participants with eating disorders, obesity and normal weight controls. Caroleo et al. (21) found significant differences among subjects with AN, binge eating disorder (BED), obesity, and normal weight controls (21). Controls had relatively higher serum IL-1α levels, but pair-wise comparisons between groups were not performed. Raymond et al. (26) found that IL-1α production by stimulated peripheral blood mononuclear cell (PBMC) from individuals with obesity was higher relative to normal weight controls, but there were no differences in IL-1α production by unstimulated PBMC among individuals with different conditions (26). INF-γ was also assessed in these two studies. Both studies found higher serum INF-γ or INF-γ production by stimulated PBMC in subjects with AN compared to normal weight controls.

IL-6 was assessed in three studies. Capuron et al. (20) found that a decrease in IL-6 one-year post bariatric surgery was associated with reduction in cognitive restraint eating accounting for variations in BMI. Raymond et al. (26) found that IL-6 production by stimulated PBMC from individuals with obesity was higher than normal weight controls, but no differences were found in IL-6 production by unstimulated PBMC. Caroleo et al. (21), on the other hand, did not identify significant differences in serum IL-6 levels among individuals with eating disorders, obesity, or normal weight.

Tumor necrosis factor (TNF)-α was assessed in three studies (21, 24, 26). Only one study with a relatively smaller sample size (N=32) found positive associations between serum TNF-α levels with binge eating scores and bulimic investigation test Edinburgh scores in a group of adolescents (24). The other two studies (N≥90) did not find significant associations between serum TNF-α levels or production by stimulated PBMC among individuals with eating disorders, obesity, or normal weight.

Other immune/inflammatory markers, including IL-1β, IL-2, IL-4, IL-7, IL-8, IL-10, transforming growth factor beta (TGF-β), monocyte chemoattractant protein 1 (MCP-1), fibrinogen, fractalkine, α-melanocyte-stimulating hormone-reactive immunoglobulin G (α-MSH/IgG) complex, and white blood cell count (WBC), were only assessed in single studies. Twenty-four-hour average plasma IL-7 levels were lower in individuals with obesity compared to those with AN restrictive type, constitutional thinness or normal weight controls (23). Serum IL-8 was positively associated with night time eating (21). IL-10 was found to be lower in individuals with obesity but higher in AN compared to normal weight controls. IL-10 levels were also associated with post-dinner eating and “not having sweet eating” (21). Serum fractalkine levels were higher in adolescent girls with obesity but lower in AN compared to healthy controls (30). Lucas et al. (25) found that plasma levels of α-MSH/IgG immune complex in individuals with obesity were lower than healthy controls (25). The melanocortin-4-receptor (MC4R) binding affinity, internalization and receptor activation of α-MSH/IgG immune complex were also decreased in individuals with obesity. On the contrary, the internalization rate of the immune complex was higher in AN. Higher white blood cell counts were found in obese individuals with BED compared to those without BED (29). Cazettez et al. (22) assessed the associations between fibrinogen and brain regions linked to eating behavior regulation (22). The authors found that higher plasma fibrinogen levels were associated with smaller lateral orbitofrontal cortex (OFC) volume in the overweight and obese group. Among individuals with excess weight, higher fibrinogen levels were linked to greater interstitial fluid in the amygdala and right parietal lobe. Other assessed inflammatory markers, including IL-1β, IL-2, IL-4, TGF-β, and MCP-1, were not significantly associated with obesity-related eating behaviors.

## Discussion

To date, only a few studies have focused on the associations between inflammatory/immune molecules and obesity-related eating behaviors. Despite the limited number of studies, a variety of inflammatory/immune markers and eating behaviors have been examined. Yet, the findings have been inconsistent. The inflammatory markers that potentially have a positive relationship with obesity-related eating behaviors include hsCRP, fractalkine, and fibrinogen. Other immune molecules, including INF-γ, IL-7, IL-10 and α-MSH/IgG, may have negative associations with obesity-related eating behaviors. However, these findings were mainly identified by single studies and replications are warranted.

Additionally, the majority of the identified studies were either case-control or cross-sectional studies, which were not capable of clarifying the causality between inflammatory markers and obesity-related eating behaviors. However, current evidence indicates a potential bi-directional relationship. Dysregulation of the immunological molecules may contribute to the development or progression of obesity-related eating behaviors. On the other hand, these eating behaviors may stimulate the production of pro-inflammatory cytokines through imbalanced nutrient intake and/or excess weight gain.

**Figure 2:**
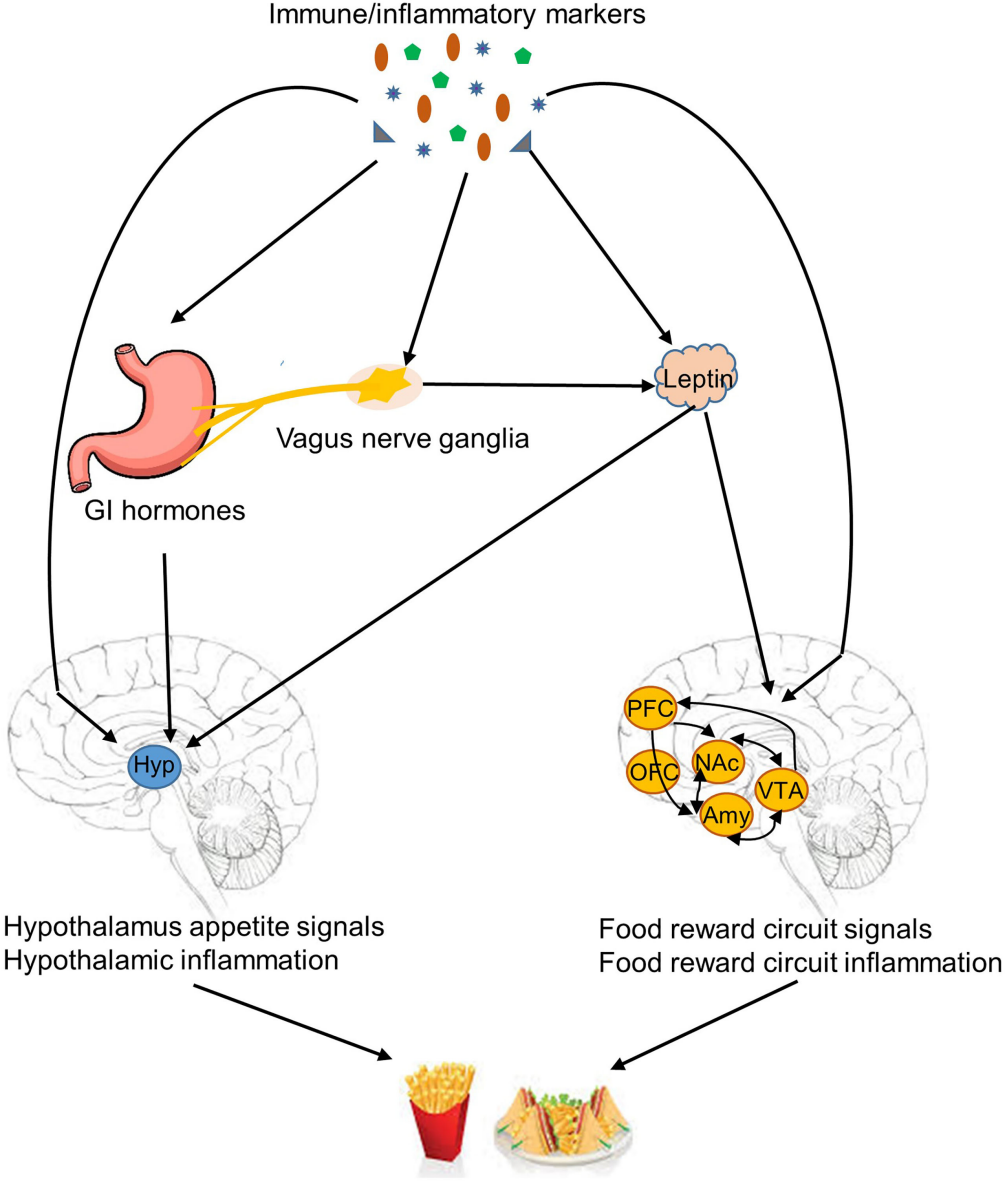
Mechanisms linked immune/inflammatory markers to obesity-related eating behaviors. Immune/inflammatory markers are potentially involved in several mechanisms of regulation of obesity-related eating behaviors, including modulation of orexigenic and anorexigenic signals in the hypothalamus, induction of hypothalamic inflammation, regulation of food reward circuitries, and suppression anorexigenic signals by induction of inflammation in the vagus nerve ganglia. GI, gastrointestinal tract; Hyp, hypothalamus; PFC, prefrontal cortex; OFC, orbitofrontal cortex; NAc, nucleus accumbens; Amy, amygdala; VTA, ventral tegmental area.

Although the blood-brain barrier prevents large molecules and cells from being freely transported to the central nervous system, studies have indicated that immune cells and cytokines with the facilitation of saturable transporters can pass through the barrier (31, 32). Also, peripheral cytokines may stimulate endothelial cells of the barrier to produce substances, such as prostaglandins, to induce inflammation and activate selective hypothalamic neurons (33, 34). Furthermore, microglia in the central nervous system share immunological functions as mononuclear cells. Both microglia and astrocytes are able to secrete inflammatory cytokines (35, 36). Once immune cells and molecules reach the central nervous system, several mechanisms potentially underlie their role in the regulation of obesity-related eating behaviors (8, 37), including modulation of orexigenic and anorexigenic signals in the hypothalamus, induction of hypothalamic inflammation, and regulation of food reward circuitries (**Figure 2**). In addition, vagus nerves may partially relay the effect of peripheral immune molecules on the regulation of obesity-related eating behaviors (38).

Evidence suggests a role of immune molecules in the regulation of orexigenic and anorexigenic signals and subsequent modulation of appetite and satiety in the hypothalamus. Inflammatory cytokines, such as IL-1β, can stimulate leptin production (15, 39). Leptin is a key adipokine that interacts with neurons in the hypothalamus and food reward circuits to modulate food intake (40). IL-6 can enhance central leptin action, increase hypothalamic signal transducer, and reduce food intake (11). Cytokines also interact with other hormones and neuropeptides involved in eating regulation. IL-1β and interferon may reduce circulating appetite-stimulating hormones, such as ghrelin, and increase appetite-suppressing hormones, such as cholecystokinin (CCK) (41–43). Cytokines, such as IL-1β and IL-7, are able to promote the expression of anorexigenic peptides, such as proopiomelanocortin, and inhibit the expression of orexigenic peptides, such as agouti-related peptide and neuropeptide Y (4, 8). Circulating immunoglobulins are also found to bind to α-melanocyte-stimulating hormone (α-MSH) to form immune complexes (α-MSH/IgG) and modulate the activation of MC4R to decrease appetite (25). Furthermore, other than the role in regulating orexigenic and anorexigenic signals, inflammatory cytokines are able to directly activate neurons in key hypothalamic appetite regulatory regions. IL-1 receptors have been found in neurons in the hypothalamic arcuate nucleus (ARC), ventromedial nucleus, paraventricular nucleus, and the lateral hypothalamic area (8). IL-7 has been shown to directly activate and improve survival of the ARC neurons (4).

Another mechanism particularly related to the perpetuation of obesity and obesity-relate eating behaviors is hypothalamic inflammation (44). In animal models, high fat feeding activates cellular inflammation in diverse tissues including the hypothalamus (5, 45). Fractalkine is involved in the recruitment of monocytic cells to the hypothalamus and promotes hypothalamic inflammation induced by high fat diet (5). Hypothalamic inflammation contributes to leptin and insulin resistance (46). Studies have shown that central inhibition of the cellular inflammatory pathway in the hypothalamus can promote leptin and insulin sensitivity, reduce high fat food intake, and consequently protect against high fat food induced obesity (47–49). IL-10, a potent anti-inflammatory cytokine, may decrease hypothalamic inflammation. Controversially, IL-10 deficiency also reduces food intake (50).

In addition to appetite and satiety regulated by hypothalamic nuclei, hedonic eating is modulated by food reward circuitries located in the nucleus accumbens, ventral tegmental area, amygdala, and OFC (40). Decarie-spain et al. (51) found that high saturated fat intake triggers inflammation in the nucleus accumbens in mice and a blockage of cellular inflammation in this brain region suppresses compulsive sucrose seeking behavior (51). The lateral OFC and amygdala are potentially involved in taste and food preference (22, 52), and are important brain regions that participate in food reward (40). Cazette et al. (22) found that elevated levels of fibrinogen, a marker of inflammation, were associated with smaller lateral OFC, and increased interstitial fluid in the amygdala and the right parietal cortex in individuals with excess weight. Therefore, inflammation in food reward circuitries may also play a role in the development or perpetuation of obesity-related eating behaviors.

Other than direct interactions between immune molecules and the central nervous system, the vagus nerve is also involved in connecting peripheral inflammatory cytokines with eating regulation (8). A high fat diet can lead to inflammation in the vagus nerve ganglia, which can attenuate the signals of appetite suppressing hormones, such as leptin and CCK, relayed by the vagus nerve to the central nervous system (38).

In addition to eating regulation by immune molecules, obesity-related eating behaviors potentially affect the serum levels of inflammatory/immune markers through accumulated adipose tissue and food intake. Eating behaviors, such as disinhibited eating, emotional eating, high intake of dietary fat, contribute to excessive calorie intake and subsequently the development of obesity (3). The hyperplasia and hypertrophy of adipose tissue in obesity produces mediators, including adipokines and fatty acids, which trigger the accumulation of macrophages and lymphocytes (53). Cytokines derived from the accumulated immune cells lead to a state of chronic, low-grade systemic inflammation associated with obesity. Therefore, obesity-related eating behaviors may contribute to the systemic inflammation, such as elevated hsCRP, through their impact on adipose tissue (54, 55). Additionally, eating behaviors directly affect dietary intake (56, 57). Diet, such as high intake of dietary fat and low intake of vegetables, and certain dietary patterns, has been linked to increased serum levels of inflammatory markers, such CRP, TNF-α, and IL-6 (58–60). Hence, obesity-related eating behaviors may alter inflammatory status through dietary intake.

Thus far, the majority of evidence on the relationship between immune molecules and obesity-related eating behaviors has been generated by animal studies. More research in humans is desirable to delineate the role of immune/inflammatory molecules in modulating obesity-related eating behaviors and subsequently become potential targets for weight loss interventions. Moving forward, more rigorous research methodologies, such as randomized controlled trials and longitudinal studies, are necessary to delineate the causal relationship between immune/inflammation markers and obesity-related eating behaviors. Additional adjustment of potential confounders will be valuable to further define this relationship (61, 62). For example, systemic inflammation and obesity-related eating behaviors usually concur with obesity. Including or adjusting BMI or fat mass in the analysis is a critical step to elucidate the relationship. Similarly, dietary intake is another potential key mediator. However, none of the included studies considered dietary intake. Future studies with sophisticated research design, comprehensive theoretical models, and robust adjustment of relevant confounders may clarify the relationship between immune/inflammation markers and obesity-related eating behaviors in humans.

## Data availability statement

The original contributions presented in the study are included in the article/supplementary material. Further inquiries can be directed to the corresponding author.

## Author contributions

YM designed the study and conducted literature searches. YM and AK screened the identified publications individually. YM summarized previous research studies and wrote the first draft of the manuscript. All authors reviewed and revised the draft and have approved the final manuscript.

## Funding

YM is funded by NINR 5 K23 NR019014-02. NINR had no role in the study design, collection, analysis or interpretation of the data, writing the manuscript, or the decision to submit the paper for publication.

## Conflict of interest

The authors declare that the research was conducted in the absence of any commercial or financial relationships that could be construed as a potential conflict of interest.

## Publisher’s note

All claims expressed in this article are solely those of the authors and do not necessarily represent those of their affiliated organizations, or those of the publisher, the editors and the reviewers. Any product that may be evaluated in this article, or claim that may be made by its manufacturer, is not guaranteed or endorsed by the publisher.
